# Lack of *C9orf72* Repeat Expansion in Taiwanese Patients with Mixed Neurodegenerative Disorders

**DOI:** 10.3389/fneur.2014.00059

**Published:** 2014-04-28

**Authors:** Chin-Hsien Lin, Ta-Fu Chen, Ming-Jang Chiu, Han-I Lin, Ruey-Meei Wu

**Affiliations:** ^1^Department of Neurology, College of Medicine, National Taiwan University Hospital, National Taiwan University, Taipei, Taiwan

**Keywords:** *C9orf72*, frontotemporal dementia, Alzheimer’s dementia, Parkinson’s disease, Parkinsonism, risk factor

## Abstract

**Background:** The hexanucleotide repeat expansion in intron 1 of the *C9orf72* gene is recognized as the most common genetic cause of frontotemporal dementia (FTD). There are overlapping clinical and pathological characteristics between FTD and Parkinsonism syndrome, and some FTD patients may present with Parkinsonism. The aim of this study was to analyze the hexanucleotide repeat numbers of *C9orf72* gene in a mixed Taiwanese cohort with FTD, Parkinsonism syndrome, Parkinson’s disease (PD), and Alzheimer’s dementia (AD).

**Method:** The number of hexanucleotide repeats was estimated in a total of 482 patients with mixed neurodegenerative disorders and 485 control subjects, using a two-step repeat-primed polymerase chain reaction-based genotyping strategy. The individual groups of patients included patients with Parkinsonism syndrome (*n* = 95), familial PD (*n* = 109), young-onset PD (*n* = 201), FTD (*n* = 9), sporadic AD (*n* = 61), and early-onset AD (*n* = 7).

**Results:** We did not identify any pathogenic repeats (>30 repeats) of *C9orf72* in either the patients or control subjects. However, we found one young-onset PD patient and one control subject that each had an intermediate number of repeats (25 and 21 repeats, respectively). The clinical phenotype of the young-onset PD in this patient was similar to typical idiopathic PD without additional features, and the patient responded well to levodopa treatment.

**Conclusion:** The repeat expansion in *C9orf72* is not a common cause of PD, Parkinsonism syndrome, or dementia in our population. Further studies are needed to investigate the clinical and biological significance of intermediate repeats in *C9orf72*.

## Introduction

A massive expansion of the GGGGCC hexanucleotide repeat in the intron between non-coding exons 1a and 1b of the chromosome 9 open reading frame 72 (*C9orf72*) gene was recently found to be a major genetic cause of familial frontotemporal dementia (FTD) and amyotrophic lateral sclerosis (ALS) (1, 2). Patients who carry the repeat expansion have an earlier onset, shorter survival, and familial aggregation of dementia or other neurodegenerative disorders than patients with normal repeat number of *C9orf72* (3). In addition to FTD and ALS, other features in patients carrying the *C9orf72* mutation can include isolated Parkinsonian movement disorder, memory impairment, and signs of cerebellar dysfunction (4). These symptoms tend to accumulate and phenotypes may converge with disease progression. Most patients eventually develop some behavioral abnormalities, as well as language and motor disabilities (5, 6).

Recently, a number of studies have found that Parkinsonism may precede, coincide, or follow the behavioral or language-predominant variant of FTD. FTD with Parkinsonism is part of a growing spectrum of the dementia–Parkinsonism continuum (7–9). The clinical phenotypes include the Parkinsonism syndrome of progressive supranuclear palsy (PSP), corticobasal syndrome (CBS), diffuse Lewy body dementia (DLBD), and typical Parkinson’s disease (PD) (10). The pathological markers of these phenotypes include TAR DNA-binding protein 43 (TDP-43), phosphorylated tau, and tau-negative but ubiquitin-positive neuronal inclusions (6, 11). Therefore, given the clinical and pathological overlaps between FTD, Parkinsonism syndrome, PD, and Alzheimer’s dementia (AD), the aim of this study was to determine whether the abnormal *C9orf72* hexanucleotide repeat expansions found in FTD patients is also associated with these other neurodegenerative disorders in a Taiwanese population.

## Materials and Methods

### Subjects

A total of 482 patients with mixed neurodegenerative disorders and 485 ethnicity matched control subjects were enrolled in the study. The individual groups included patients with Parkinsonism syndrome (*n* = 95), familial PD (*n* = 109), young-onset PD (age at onset <50 years, *n* = 201), FTD (*n* = 9), sporadic late-onset AD (*n* = 61), and early-onset AD (age at onset <50 years, *n* = 7). Mutations in the *a-synuclein, Parkin, PINK1, DJ-1, LRRK2, SCA2, SCA3, ATP13A2*, and *HTRA2* genes were excluded in all young-onset PD patients (12–16).

Patients with PD were diagnosed using the UK brain bank diagnosis criteria (17). Patients with FTD were diagnosed using standard criteria by the Work Group on FTD and Pick’s Disease (18). AD patients were diagnosed by the NINCDS-ADRDA criteria (19). Unrelated adult volunteers without neurological disease were recruited as controls from the community and from our hospital. Informed consent was given by all study participants, and the study was approved by the institutional ethics board committees of National Taiwan University Hospital.

### Genetic analysis

DNA was extracted from venous blood using standard protocols (12). The size of the hexanucleotide repeats in the *C9orf72* alleles was detected using a two-step repeat-primed polymerase chain reaction-based genotyping strategy, as previously described (1).

### Statistical analysis

Descriptive statistics were expressed as the mean ± the standard deviation. Differences in the distributions of repeat number between the individual patient groups and controls were tested using a two-tailed Mann–Whitney *U*-test and significance was set at *p* = 0.05. Statistical analysis was performed using the STATA, version 8.0.

## Results

The demographic data of all tested subjects are summarized in Table 1. We used a previously suggested cutoff (>30 repeats) to distinguish the pathogenic expansion from the normal allele (2). We did not detect any pathological repeat expansions of *C9orf72* in either patients or control subjects.

**Table 1:** **Demographic data of the enrolled subjects in each disease group**.

Subject group	Patient number (male/female)	Current age (years)	Onset age (years)
Parkinsonism syndrome	95 (53∕42)	69.1 ± 13.3	59.5 ± 13.6
MSA	51 (26∕25)	69.5 ± 12.7	60.1 ± 12.5
PSP	14 (9∕5)	69.1 ± 12.9	60.2 ± 12.6
CBS	2 (2∕0)	68.9 ± 13.3	57.6 ± 13.8
PDD	17 (10∕7)	69.6 ± 13.1	60.2 ± 12.7
DLBD	3 (2∕1)	69.9 ± 12.3	59.1 ± 12.8
Parkinsonism with dystonia	6 (3∕3)	34.3 ± 13.6	18.7 ± 12.1
Parkinsonism with FTD	2 (1∕1)	69.5 ± 7.5	65.3 ± 11.5
Familial PD	109 (57∕52)	66.5 ± 12.2	55.6 ± 13.4
Young-onset PD	201 (103∕98)	54.3 ± 7.6	42.5 ± 5.2
Dementia	77 (29∕48)	58.1 ± 11.4	55.0 ± 4.5
FTD	9 (4∕5)	58.0 ± 13.9	53.9 ± 7.8
AD	61 (20∕41)	64.1 ± 4.5	55.0 ± 4.5
Early-onset AD	7 (6∕1)	50.6 ± 4.2	46.7 ± 3.2
Control subjects	485 (233∕252)	60.6 ± 11.9	N.A.

*The data are shown as mean ± standard deviation*.

MSA, multiple system atrophy; PSP, progressive supranuclear palsy; CBS, corticobasal syndrome; PDD, Parkinson’s disease with dementia; DLBD, dementia with Lewy bodies; FTD, frontotemporal dementia; PD, Parkinson’s disease; AD, Alzheimer’s dementia; N.A., not applicable

**Figure 1:**
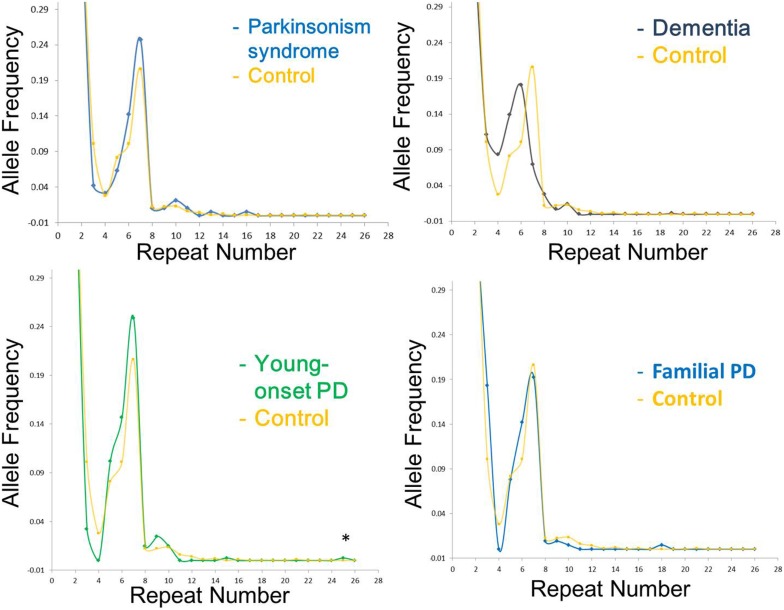
**Distribution of C9orf72 (C4G2) repeat number in different disease group as compared to control subjects in the current study**. The *X*-axis represents the estimated repeat number, whereas the *Y* -axis represents the allele frequency. Asterisk indicates intermediate repeats (20–29 repeats).

The range of repeat expansions detected in our participants was 2–25 units, and the most frequent repeat number was 2 repeats, followed by 6, 7, and 5 repeats. Notably, we found one young-onset PD patient who had an intermediate number of 25 repeats, and one control subject who harbored 21 repeats (Table 2). The clinical phenotype of the young-onset PD in this patient was similar to typical idiopathic PD without additional features, and the patient responded well to levodopa treatment. The distribution of the estimated repeat numbers for each disease group compared to control subjects is presented in Figure 1.

**Table 2:** **Distribution of *C9orf72* (GGGGCC) repeat numbers in each disease group and control subjects**.

*C9orf72* (GGGGCC) repeat number	Parkinsonism syndrome (*n* = 190 alleles) *n* (%)	Familial PD (*n* = 218 alleles) *n* (%)	Young-onset PD (*n* = 402 alleles) *n* (%)	Dementia (*n* = 154 alleles) *n* (%)	Control subjects (*n* = 970 alleles) *n* (%)
1	0	0	0	0	0
2	78 (41.1)	81 (37.2)	165 (41.0)	52 (33.8)	416 (42.9)
3	8 (4.2)	39 (17.9)	13 (3.2)	26 (16.9)	98 (10.1)
4	6 (3.1)	2 (0.9)	0	12 (7.8)	27 (2.8)
5	12 (6.3)	17 (7.8)	41 (10.2)	20 (13.0)	79 (8.2)
6	27 (14.2)	31 (14.2)	59 (14.6)	26 (16.9)	98 (10.1)
7	47 (24.7)	42 (19.2)	100 (24.9)	10 (6.5)	200 (20.6)
8	2 (1.1)	2 (0.9)	6 (1.5)	4 (2.6)	12 (1.2)
9	2 (1.1)	2 (0.9)	10 (2.5)	1 (0.6)	12 (1.2)
10	4 (2.1)	1 (0.5)	6 (1.5)	2 (1.3)	13 (1.4)
11	2 (1.1)	0	0	0	6 (0.6)
12	0	0	0	0	4 (0.4)
13	1 (0.5)	0	0	0	1 (0.1)
14	0	0	0	0	2 (0.2)
15	0	0	1 (0.3)	0	0
16	1 (0.5)	0	0	0	1 (0.1)
17	0	0	0	0	0
18	0	1 (0.5)	0	0	0
19	0	0	0	1 (0.6)	0
20	0	0	0	0	0
21	0	0	0	0	1 (0.1)^a^
22	0	0	0	0	0
23	0	0	0	0	0
24	0	0	0	0	0
25	0	0	1 (0.3)^a^	0	0
26	0	0	0	0	0

*The data are shown as number (percentage); PD, Parkinson’s disease*.

*^a^Intermediate repeats (20–29 repeats)*.

## Discussion

Numerous studies have supported the concept that various neurodegenerative disorders share overlapping clinical, genetic, and pathologic features. Although the clinical manifestations vary, some patients with FTD can also show features of PD or Parkinsonism syndromes, characterized as PSP, CBS, or DLBD. The identification of a hexanucleotide repeat expansion in the *C9orf72* gene as a frequent cause of both FTD and ALS suggests a possible role of this genetic alteration in other dementias or Parkinsonism-related movement disorders. Herein, we demonstrate that the *C9orf72* repeat expansion is not a common cause of disease for sporadic AD, FTD, PD, and other related movement disorders in our population.

Given the relatively small sample size of the individual patient groups in our series, we cannot definitively exclude the possibility of a role for the *C9orf72* repeat expansion in disease risk. In parallel with our findings, other recent studies have also shown an absence of an association between repeat expansion in the *C9orf72* gene and disease susceptibility in AD, PD, or related Parkinsonism syndromes, especially in the Asian population (20–24). We speculate that one of the possible reasons why no expansions have been found in our cases or controls may be due to the fact that carriers of the repeat expansion at *C9ORF72* arose from a European single common founder, implying that expansions are rare in non-Caucasian populations (25). Another possible reason is that we did not enroll any patients with ALS and the case number in the FTD group in small in our present study. Therefore, although the abnormal repeats of *C9orf72* gene account for 23–47% of familial FTD with or without ALS and 4–20% of sporadic ALS (2, 21, 26), our observation combined with previous findings suggest that variation in the *C9orf72* does not play a major role in the susceptibility to the wider spectrum of Parkinsonism and dementia syndromes. Pathological expansion of the *C9orf72* hexanucleotide repeats may be specific to TDP-43 pathology-associated FTD and ALS.

Based on the allele frequencies in cases and controls, the first studies suggested that expansions with more than 30 repeats should be considered pathological, while alleles with <20 repeats are wild type (2). However, subsequent reports have found that some control subjects may have repeat numbers of 20–30, with 23 being the most frequently reported maximum repeat number (1, 26). Hence, the contribution of the intermediate-size alleles (20–29 repeats) to disease pathology needs to be further evaluated. Notably, recent studies demonstrated that intermediate expansions of the hexanucleotide repeats in *C9orf72* may associate with an increased risk of PD, especially those with repeat numbers over 23 (10, 20, 27–29). In our study, we identified one young-onset PD patient harboring a repeat number of 25. The mechanism by which this intermediately expanded repeat number may cause this disease pathophysiology remains unclear. One hypothesis is that the expanded hexanucleotide repeat may bind to other RNAs, resulting in protein sequestration from normal processing and then decreased protein expression (2, 21). Future functional studies are necessary to elucidate the role of intermediate repeats in *C9orf72* in neuronal degeneration.

In conclusion, our results do not suggest that an expanded repeat number in the *C9orf72* gene plays a major role in the susceptibility to the wider spectrum of movement disorders. Further large-scale studies are required to investigate the clinical and biological significance of intermediate repeats in the *C9orf72* gene in degenerative neurological disorders.

## Conflict of Interest Statement

The authors declare that the research was conducted in the absence of any commercial or financial relationships that could be construed as a potential conflict of interest.
